# Editorial: Antibiotics overuse as the driving force behind antimicrobial resistance

**DOI:** 10.3389/fcimb.2024.1373846

**Published:** 2024-02-29

**Authors:** Liran Shani, Israel Nissan

**Affiliations:** ^1^ Memed Diagnostics Ltd, Haifa, Israel; ^2^ National Public Health Laboratory, Public Health Directorate, Ministry of Health, Tel Aviv, Israel; ^3^ Division of Avian Diseases, Kimron Veterinary Institute, Veterinary Services, Beit Dagan, Israel

**Keywords:** antibiotics resistance, antibiotics stewardship, AMR (antimicrobial resistance), resistant bacteria, antibiotics overuse

Antibiotics are among the most important discoveries in medicine, saving millions of lives and improving quality of life for countless people. unfortunately, the misuse of these drugs in human, veterinary and agricultural settings have led to the emergence and spread of antimicrobial resistance (AMR).

According to the World Health Organization (WHO), Antimicrobial resistance (AMR) is one of the top global public health and development threats. It is estimated that bacterial AMR was directly responsible for 1.27 million global deaths in 2019 and contributed to an additional 4.95 million deaths[1]. In addition to death and disability, AMR has significant economic costs. The World Bank estimates that AMR could result in US$ 1 trillion additional healthcare costs by 2050, and US$ 1 trillion to US$ 3.4 trillion gross domestic product (GDP) losses per year by 2030 [2].

AMR occurs when microorganisms such as bacteria, fungi, viruses, and parasites evolve mechanisms to withstand the effects of antibiotics and other antimicrobials, making infections harder to treat and increasing the risk of disease transmission, morbidity, and mortality.

It is crucial to understand the various conditions and mechanisms of AMR development to advance research aimed at identifying and preventing the consequences of AMR.

This Research Topic brings together five papers that explore different aspects of antibiotic overuse and its impact on AMR. The papers cover a wide range of topics, including physician perceptions of antibiotic use, risk factors for colonization with multidrug-resistant bacteria, antibiotic resistance in *Mycoplasma genitalium*, the impact of perioperative antimicrobial eye drops on the ocular surface microbiome, and the genomic characterization of *Mycobacterium tuberculosis*.

In their paper, Ghoshal et al. evaluated the Physician’s perceptions on the use of antibiotics and probiotics in adults. This paper presents the results of an international survey conducted among physicians in seven countries in Asia-Pacific area, exploring their knowledge, attitudes, and practices regarding the use of antibiotics and probiotics in adults. The paper reveals significant gaps and variations in physician awareness and adherence to antibiotic stewardship guidelines, as well as low confidence and familiarity with probiotics as a potential strategy to prevent or treat antibiotic-associated adverse events.

Azzini et al. investigated the prevalence and risk factors for colonization with multidrug-resistant Gram-negative bacteria (MDR-GNB) and Clostridioides difficile (CD) among residents of long-term care facilities (LTCFs) in Italy, a high endemic setting for AMR. The paper identifies several factors associated with increased risk of colonization. Of note, the presence of a medical device and previous antibiotic use were significantly associated with 3rd-generation cephalosporin resistant GNB colonization, while the presence of a medical device and previous hospitalization were significantly associated with carbapenem-resistant (CR) GNB.

Looking at the geographic behavior of *Mycobacterium tuberculosis* (MTB) in Israel, Losev et al. characterized the genetic diversity, drug susceptibility profile, and demographic features of MTB, the causative agent of tuberculosis (TB), in Israel in 2021. The paper uses whole genome sequencing (WGS) and phenotypic testing to analyze 156 MTB isolates from laboratory-confirmed TB patients, revealing a high level of genetic heterogeneity and a low proportion of multidrug-resistant (MDR) and extensively drug-resistant (XDR) strains. The paper highlights migration as a source of MTB introduction and transmission in Israel, influenced by the demographic makeup of the State of Israel.

The final two papers demonstrated AMR dynamics in tissue-specific environment. Sandri et al. described the molecular epidemiology and antibiotic resistance patterns of *Mycoplasma genitalium* (MG), a sexually transmitted bacterium that causes urethritis, cervicitis, pelvic inflammatory disease and infertility. The paper analyzed genital and extragenital samples from men-who-have-sex-with-men (MSM) attending a sexually transmitted infection (STI) clinic in Italy, finding high rates of resistance-associated mutations to macrolides and fluoroquinolones, the first- and second-line treatments for MG infection, according to the European guidelines. The paper highlights the importance of using resistance-guided therapy as the best approach for achieving treatment success. Hotta et al. examined the impact of perioperative antimicrobial eye drops on the ocular surface microbiome in patients undergoing cataract surgery. The paper showed that the use of these eye drops alters the diversity and composition of the ocular microbiota, potentially affecting ocular health and immunity. In some patients, the microbiome did not return to its original composition, even 12 weeks after surgery.

Cumulatively, these documents highlight the intricate nature and pressing nature of antimicrobial resistance (AMR), underscoring the imperative for interdisciplinary and cooperative initiatives to address this worldwide predicament. The perspectives and suggestions presented in these documents strive to enhance the judicious utilization of antibiotics and other antimicrobials, amplify surveillance and diagnostic capabilities, innovate novel therapeutic and preventative measures, and foster awareness and education among both healthcare practitioners and the broader public. It is our expectation that this research focus will play a role in advancing scientific understanding and catalyzing additional investigations into AMR.

## Author contributions

LS: Writing – original draft, Writing – review & editing. IN: Writing – original draft, Writing – review & editing.

